# Monitoring lineages of growing and dividing bacteria reveals an inducible memory of *mar* operon expression

**DOI:** 10.3389/fmicb.2023.1049255

**Published:** 2023-06-20

**Authors:** Calin C. Guet, Luke Bruneaux, Panos Oikonomou, Maximino Aldana, Philippe Cluzel

**Affiliations:** ^1^Institute for Biophysical Dynamics and the James Franck Institute, The University of Chicago, Chicago, IL, United States; ^2^Molecular and Cellular Biology Department and John A. Paulson School of Engineering and Applied Sciences, Harvard University, Cambridge, MA, United States; ^3^Institute of Science and Technology Austria, Klosterneuburg, Austria; ^4^Department of Biological Sciences, Columbia University, New York, NY, United States; ^5^Instituto de Ciencias Físicas and Centro de Ciencias de la Complejidad, Universidad Nacional Autónoma de México, Cuernavaca, Mexico

**Keywords:** single cell, gene expression, bacteria, heterogeneity, non genetic memory

## Abstract

In Gram negative bacteria, the *m*ultiple *a*ntibiotic *r*esistance or *mar* operon, is known to control the expression of multi-drug efflux genes that protect bacteria from a wide range of drugs. As many different chemical compounds can induce this operon, identifying the parameters that govern the dynamics of its induction is crucial to better characterize the processes of tolerance and resistance. Most experiments have assumed that the properties of the *mar* transcriptional network can be inferred from population measurements. However, measurements from an asynchronous population of cells can mask underlying phenotypic variations of single cells. We monitored the activity of the *mar* promoter in single *Escherichia coli* cells in linear micro-colonies and established that the response to a steady level of inducer was most heterogeneous within individual colonies for an intermediate value of inducer. Specifically, sub-lineages defined by contiguous daughter-cells exhibited similar promoter activity, whereas activity was greatly variable between different sub-lineages. Specific sub-trees of uniform promoter activity persisted over several generations. Statistical analyses of the lineages suggest that the presence of these sub-trees is the signature of an inducible memory of the promoter state that is transmitted from mother to daughter cells. This single-cell study reveals that the degree of epigenetic inheritance changes as a function of inducer concentration, suggesting that phenotypic inheritance may be an inducible phenotype.

## Introduction

**Figure 1:**
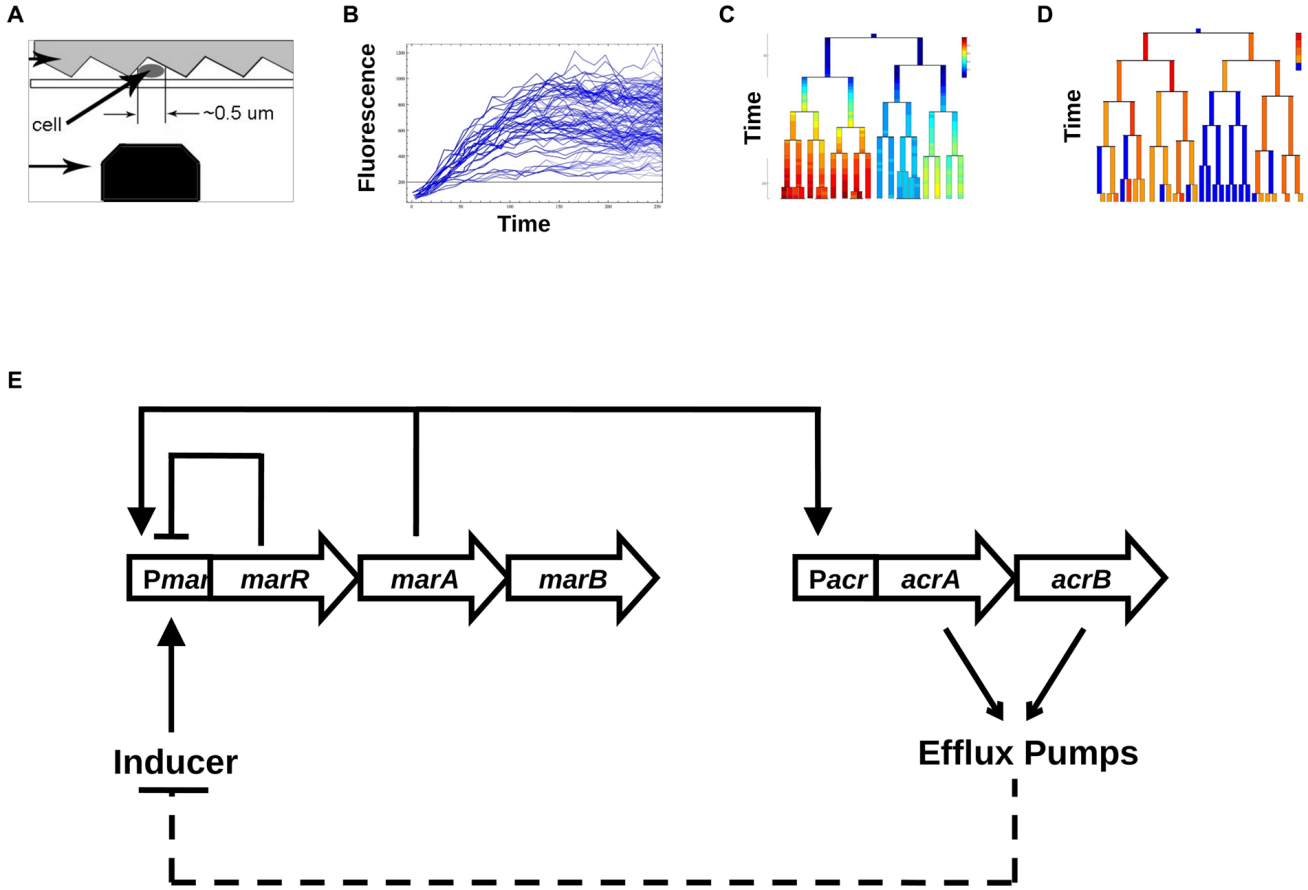
Schematic map of our experimental and analysis approach. **(A)** Data collection. Single *Escherichia coli* cells are trapped between a patterned agarose pad and a cover slip, and are excited via a blue laser that moves along the length of the cell using a scanning nano-positioning stage. **(B)** Conversion into time series of total fluorescence. From the raw data, time series of fluorescence are produced across growing colonies. **(C)** Changes in [YFP] as a function of time across generations of growing and dividing bacteria. **(D)** Binarization of promoter activities, into active and inactive cells. **(E)** The core transcriptional network of the *mar* system includes the repressor MarR that regulates the *marRAB* operon. In the presence of the inducer salicylate, MarR repression is abolished, allowing MarA to activate its own expression and the expression of the *acrAB* operon. The *acrAB* genes form the efflux pump complex AcrAB-TolC. Efflux pumps create a negative feedback on *marRAB* activity due to efflux of the inducer salicylate.

Advances in time-lapse microscopy (Locke and Elowitz, 2009) and substrate-printing (Bennett and Hasty, 2009), combined with new fluorescent reporter proteins, have facilitated the characterization of stochastic phenotypic behaviors (Cluzel et al., 2000; Elowitz and Leibler, 2000; Le et al., 2005; Le et al., 2006; Guet et al., 2008; Chang et al., 2022) that are often masked by population measurements (Balaban et al., 2004). Most of these methods depend upon measuring exclusively the concentration of fluorescent reporters. The accumulation and dilution of these reporters obscure time-dependent fluctuations at short timescales. These fluctuations are less of a limitation at long timescales in studies of bistable systems such as the *ara* and *lac* operons (Novick and Weiner, 1957; Siegele and Hu, 1997; Vilar et al., 2003) or bacterial persistence (Balaban et al., 2004), in which positive feedback loops lead to clearly distinct “on” and “off” states (i.e., digital networks). However, most gene regulatory networks produce a continuously varying distribution of outputs (i.e., analog networks), and thus detecting the transmission of information from cell to cell especially at cell division can be very difficult (Austin et al., 2006; Taniguchi et al., 2010). In order to detect inheritance in networks with analog outputs, we have developed a technique using promoter activity time-series in single-cell lineages to quantify epigenetic inheritance for a gene regulatory network (Figures 1A–D). We use the promoter of the *m*ulti*-a*ntibiotic *r*esistance (*mar*) operon from *Escherichia coli* fused to a gene coding for a fluorescent protein to test our technique. The study of single cell lineages reveals the presence of an inducible inheritance phenotype in the expression of the *mar* promoter as measured from a very low-copy reporter plasmid.

The *mar* operon also denoted as *marRAB*, consists of three genes: *marR*—a repressor, *marA*—an activator and *marB*—whose function is not fully understood. The biology of the *mar* operon has been extensively studied at the population level (Martin et al., 1996), yet little is known about the dynamical aspects of this inducible gene regulatory network at the single-cell level. Through the direct and indirect action of MarA on about 60 different genes, *mar* represents one of the largest regulons of *E. coli* (Barbosa and Pomposiello, 2005). One of the main targets of MarA is activation of the expression of the multi-drug resistance efflux pump operon *acrAB* (Li and Nikaido, 2004). In the absence of an inducer, the *mar* network is characterized by a negative feedback loop produced by MarR self-repressing the *mar* operon (Alekshun and Levy, 1997; Figure 1E). In the presence of an inducer, such as salicylate, MarR repression is relieved, and the activator MarA initiates its own expression, constituting a positive feedback loop. MarA also activates the *acrAB* and *tolC* operons, that together encode the AcrAB-TolC efflux pumps (Martin et al., 2008), the main multi-drug resistance determinant of Gram negative bacteria. Over time, the accumulation of efflux pumps reduces the intracellular concentration of inducer, which constitutes a negative feedback loop by renewing the repression of the *mar* operon by MarR. Thus, the activation of the *mar* system confers multi-drug resistance (George and Levy, 1983) that is eliminated when the pumps are no longer expressed (Alekshun and Levy, 1997; Le et al., 2006). The reversible nature of this resistance suggests that individual cells may have widely ranging responses to a steady input (Lewis, 2007). Interestingly, the alternating behavior of the positive and negative feedback loops (Figure 1E) has the potential to reverse the activation of the *mar* promoter even under a steady level of inducer (Thomas and Richelle, 1988; Le et al., 2006; Aracena, 2008).

Here, we measured activity of the *mar* promoter in single cells using a confocal scanning volume across *E. coli* colonies growing as linear micro-colonies, starting from a single “mother” cell. The *mar* promoter controls the expression of a *yfp-venus* reporter with very fast maturation time (Balleza et al., 2018) expressed from a very low-copy plasmid with SC101* *ori* (Lutz and Bujard, 1997). Using the promoter activity reported via YFP expression, we constructed genealogy trees describing promoter induction for individual lineages of *E. coli*. We used an algorithmic process to binarize the output of the promoter and then calculated a measure for the degree of epigenetic inheritance of each individual lineage at various inducer concentrations. Surprisingly, we found that the coefficient of variation (CV) of expression depended in a non-monotonic way on inducer concentration. Based on our analysis, we found that the degree of epigenetic inheritance changed as a function of inducer concentration, suggesting that phenotypic inheritance may be an inducible phenotype within an analog-output gene regulatory network.

## Materials and methods

### Strains and plasmids

We used the *E. coli* K-12 strain Frag1B (Le et al., 2005) for all experiments. Plasmid pZS*1mar-venus: We replaced the *gfp* from pZS*1*R*-*gfp* (Guet et al., 2002) with the *venus-yfp* gene using KpnI and HindIII sites. We PCR amplified the *mar* promoter, containing the marbox and the two MarR operators, from the Frag1B chromosome. Since one operator for MarR overlaps with the *marR* coding region, we retained the first seven amino acids at the N-terminus of MarR and fused them to Venus-YFP (Martin and Rosner, 2004). We mutated the wild-type GTG start codon of *marR* to ATG by changing G with A in the primer. We cloned the *mar* promoter into the XhoI and KpnI sites of pZS*1*R*-*venus*. The KpnI site introduced a Gly-Thr linker between MarR and Venus-YFP. The pZS*1*mar-venus* plasmid contains Amp^R^ and has a SC101* *ori* that maintains the copy number at 3–4 copies/cell (Lutz and Bujard, 1997). We transformed all strains by electroporation.

### Growth conditions

We grew cells overnight at 30°C in Luria-Bertani broth (LB) containing 100 μg/mL ampicillin (Sigma Aldrich, St. Louis, MO, United States). We diluted the overnight cultures 1:600 in fresh LB media and harvested cells after an additional 3.5 h at 30°C. The optical density (OD) of the final cultures varied between 0.12–0.15 as measured at 600 nm.

### Sample preparation for microscopy and submicron-groove fabrication

We prepared fresh 3% low melting agarose (Fisher Scientific, Pittsburgh, PA, United States) in LB in a 70°C water bath. We mixed salicylate from a 1 M sodium salicylate (Fluka, Switzerland) stock solution with 100 μL LB and added the mixture to 5 mL LB-agarose and swirled vigorously for 1 min. in order to achieve rapid and homogeneous mixing. We poured 100 μL of this 3% agarose-LB as a 1 cm^2^ gel slab onto an optical grating (300 lines/mm, blaze angle 26°, Bausch and Lomb, Rochester, NY, United States). The gel pad solidified for 20 min in the dark at room temperature, imprinting the grooves of the optical grating in the gel. We placed 0.3 μL of freshly harvested *E. coli* cells on top of a thin coverslip (Fisher Scientific, United States) sealed with wax to an aluminum holder. We placed the agarose-LB slab on top of the 0.3 μL droplet of cells. Due to surface tension, most cells aligned themselves in the submicron-grooves. The sample was sealed and immediately placed on the heating stage of an inverted Olympus X71 microscope (Olympus, Japan). We began collecting data within 5–10 min of the sample set up.

### Number of cells measured

For each salicylate inducer condition we measured 10 different linear micro-colonies. Experiments were carried out over different days. For 0 mM we measured 392 mother-daughter pairs, for 0.25 mM 430 pairs, for 1 mM 510 pairs, for 3 mM 457 pairs.

### Data collection and processing

To measure fluorescence from individual cells, we focused blue laser light (Sapphire 488 nm, 20 mW, Coherent, Santa Clara, CA, United States) into a diffraction-limited spot (width = 0.4 μm; Guet et al., 2008) and scanned cells at 4 μm/s using a nano-positioning stage (Physik Instrumente, Auburn, MA, United States). Emitted photons were collected in a confocal geometry and detected with an avalanche photodiode (ALV, Langen, Germany). This scanning procedure is minimally invasive and accurately measures fluorescence intensity within individual cells (Guet et al., 2008). We scanned each cell every 5–10 min. We aligned the scanning trajectory of the laser spot perfectly along the submicron-grooves using the nano-positioning stage. Fluorescence data was stored for further analysis.

Each scan produced a fluorescence profile of individual cells from a micro-colony. Fluorescent data was binned into 0.4 μm pixels. Because the excitation spot was small (width = 0.4 μm), cell cleavage sites appeared as sharp dips in the fluorescence profile while cell bodies appeared as long, bright fluorescence plateaus. Cell length was the distance between two consecutive cleavage sites. The difference in length between two consecutive measurements was used to estimate the cell growth rate. To produce genealogy trees, we used a visual interface (in house software, Matlab). Using this program, we manually picked cleavage sites from the intensity profiles produced by the scanning and assigned and tracked cell lineages. The intracellular concentration of fluorescent proteins was given by the brightest pixel. The fluorescence intensity was directly related to the intracellular concentration of fluorescent molecules. The promoter activity was the change in fluorescence intensity between two time points corrected for by a dilution factor that accounts for growth. We assigned each cell a single value for promoter activity between divisions.

### Data analysis

#### Binarization

We binarized promoter activity as either ON (active) or OFF (inactive) using a threshold. Activity was ON if the cell had promoter activity at least two standard deviations above the mean of the distribution of activities of un-induced cells. Activity was OFF if the promoter activity was below this threshold.

#### Domain size and degree of inheritance

For each tree, we defined a sub-tree as two or more cells that were directly connected (through mother-daughter or sister–sister relationships) and all shared the same promoter activity, i.e., ON or OFF. For each tree, we computed the mean sub-tree size of ON and OFF sub-trees, *<ON >* and *< OFF>*, respectively. The mean sub-tree size is a measure of how large the average sub-tree is in which a randomly selected cell finds itself on a genealogy tree. It is defined as:

$$\langle ON\rangle =\frac{\sum_{S}\;n_{S}^{on}s^{2}}{\sum_{S}\;n_{S}^{on}s},$$

where $n_{S}^{on}$ is the number of ON domains that contain *s* cells, and represents the definition of a mean domain size in percolation theory (Stauffer and Aharony, 1994). For each tree we calculated the product *<ON > <OFF>*. For a given inducer concentration, the degree of inheritance was defined as the average domain size product, or ∑〈ON〉〈OFF〉 divided by the number of trees, *N*.

### Computational model

We implemented a discrete-time branching process in which all cells in the population divide simultaneously. Each cell can be in one of two possible states: active (on) or inactive (off). When a cell divides, each of its two daughter cells can be active or inactive depending on the state of the mother cell. To implement the model, four conditional probabilities must be defined. Denoting as DC and MC the daughter and mother cells, respectively, the four conditional probabilities are: P(DCon|MCon) , P(DCoff|MCon) , P(DCon|MCoff) , and P(DCoff|MCoff) . Only two of these four probabilities are independent since, P(DCon|MCon)=1−P(DCoff|MCon) , and P(DCoff|MCoff)=1−P(DCon|MCoff) . Therefore, the complete branching process is thoroughly characterized by the two probabilities P(DCoff|MCon) and P(DCon|MCoff) . These two probabilities were computed from experimental data for different inducer concentrations. Once the relevant probabilities are fixed, the branching process starts with one cell in the “on” state assuming that this cell has been placed in a medium with nonzero inducer concentration. From there, the branching process evolves.

## Results

### Measuring marRAB promoter induction in individual bacteria across linear micro-colonies

We cloned the full length *mar* promoter upstream of Venus *yfp* (Nagai et al., 2002), on a very low copy plasmid with SC101* origin (3–4 copies per cell; Lutz and Bujard, 1997).

**Figure 2:**
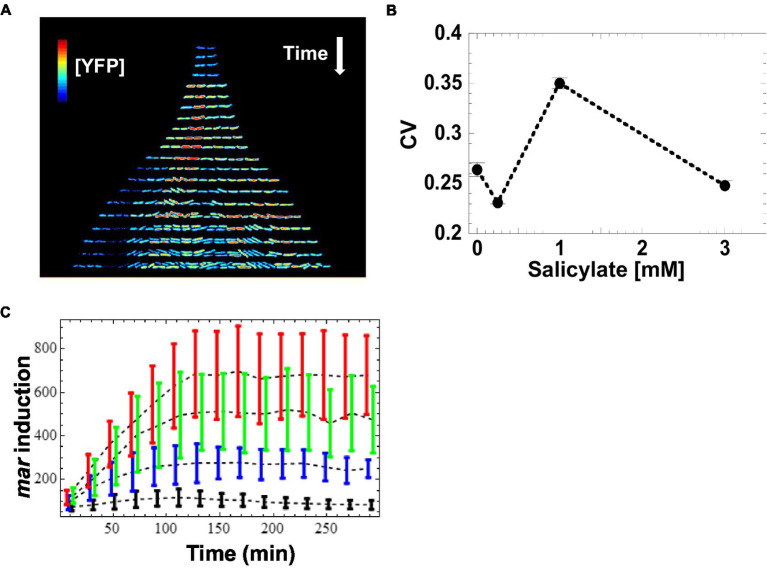
Induction of *mar* promoter with salicylate. **(A)** Induction of the *mar* promoter in individual cells of a linear colony. Single colony images are separated by ~20 min. The heat map shows fluorescence intensity (Red = High, Blue = Low) from YFP expression driven by the *mar* promoter activity in individual cells exposed to a steady level of salicylate (1 mM). Induction starts at *t* = 9 min. **(B)** Mean coefficient of variation of [YFP]. YFP fluorescence from single cells was averaged for 10-min intervals and the coefficient of variation across the interval was calculated. The mean coefficient of variation across the interval 120 to 300 min was then calculated. Error bars represent the standard error. **(C)** Induction kinetics of the *mar* promoter at the population level. YFP fluorescence from single cells was averaged for 10-min intervals, reporting *mar* promoter activity as a function of time. Four different salicylate concentrations were used: 0 mM (black), 0.25 mM (blue), 1 mM (green), 3 mM (red). Error-bars are the standard deviation from the distribution of single cell measurements.

The SC101* origin has a very tight regulation of partitioning. Consequently, partitioning governed by SC101* origin is accompanied by much lower plasmid noise than that of higher copy number plasmids (e.g., colE1 origin) that rely on unregulated random partitioning. The fast maturation of the Venus-YFP chromophore allows us to report (Balleza et al., 2018) changes in *mar* promoter activity within a few minutes. We monitored salicylate-induced expression of the *mar* promoter as a function of time in wild-type *E. coli* cells at the single cell-level across several inducer concentrations that cover the dynamic range of previous population measurements (Rosner, 1985; Cohen et al., 1993). To limit cell–cell contact, we restricted cell growth to one dimension by growing bacteria as linear colonies within long micro-grooves imprinted into agar. This geometry ensures that all bacterial cells are grown in similar micro-environmental conditions. To ensure the genetic and epigenetic homogeneity of a linear colony during growth, we restricted our measurements to micro-grooves that were initially occupied by only a single bacterium. By scanning the colonies with a focused blue laser and collecting the emitted fluorescence with a photodiode (Guet et al., 2008), we monitored the expression level of YFP in individual bacteria exposed to a steady level of the inducer salicylate ranging from 0 to 3 mM. We measured the concentration of the YFP reporter, [YFP], at 5–10 min intervals in individual cells across the linear micro-colonies. At the single-cell level, we observed that [YFP] varied several-fold within colonies, even between siblings (Figure 2A). Using the coefficient of variation (CV) as a quantitative estimate of the cell-to-cell variability, we found the highest variability in [YFP] at 1 mM salicylate (CV = 0.35 at 1 mM; CV ≤ 0.25 for the other inducer concentrations; Figure 2B). By contrast, the average [YFP] across all colonies reached a well-defined steady state for each concentration of inducer about 120 min after induction (Figure 2C).

### Monitoring gene expression across generations

Given the increase in CV value for the intermediate inducer value (1 mM) we observed, we wanted to find out whether this variation was a result of random fluctuations in individual bacteria, or if this effect was due to a transmission of cytoplasmic information from a mother cell to its daughters. To characterize how *mar* promoter gene expression changed through cell lineages, we monitored [YFP] across generations within the linear colonies and we represent the changes in activity using genealogy trees (Figure 2A). We generated the trees by presenting the time series of [YFP] concentration for a single lineage under each inducer level, with the concentration of [YFP] denoted from low (blue) to high (red). Both, the absence of salicylate (0 mM) and high (3 mM) concentration of salicylate led to homogeneous [YFP] across several generations of bacteria grown from an initial mother cell (Figure 2A; Supplementary Figures S1A,G). Remarkably, an intermediate salicylate concentration (1 mM) resulted in heterogeneous distribution of [YFP], ranging from very high (as observed with 3 mM salicylate) to very low (as observed with no salicylate; Figure 2A; Supplementary Figure S1E). The observed heterogeneity in gene expression forms sub-trees with high and low levels of [YFP] that coexist within a micro-colony, which is suggestive of an inherited gene expression state and which helps explain the much higher CV levels observed in Figure 2B.

### Binarization of promoter activity and quantification of inheritance

**Figure 3:**
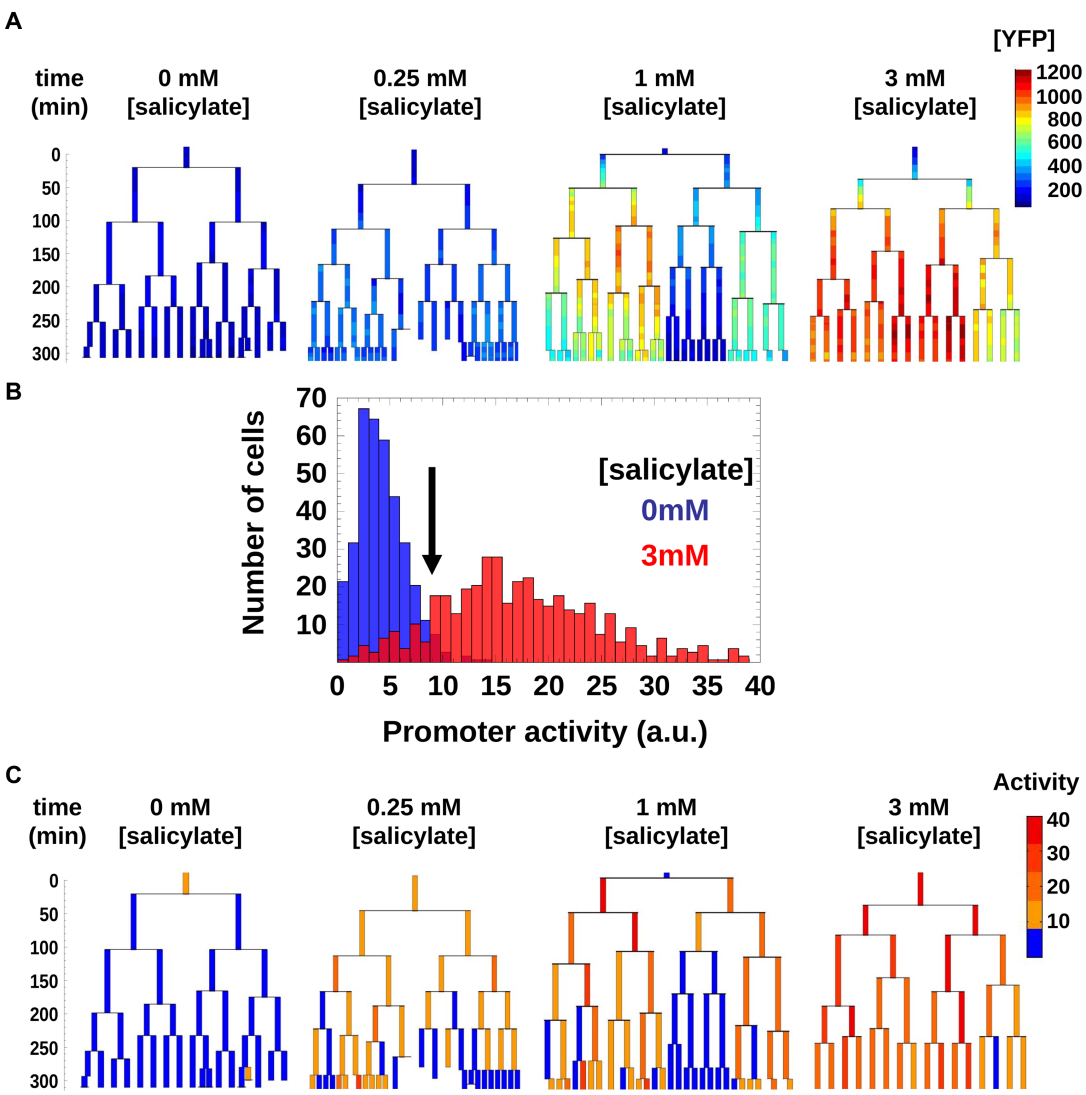
Genealogy trees of *mar* promoter induction in wild-type cells. **(A)** Single cells with a plasmid carrying *yfp-venus* regulated by the *mar* promoter were monitored through several generations in the presence of the inducer salicylate. The genealogy tree follows the induced level of YFP concentration through the generations of a colony. Constant salicylate exposure began at time *t* = 0. Heat-map scale uses arbitrary fluorescence units to show low (blue) to high (red) [YFP]. **(B)** Distribution of *mar* promoter activity across a population of non-induced 0 mM salicylate (blue) and fully induced 3 mM salicylate (red) cells. The promoter activity is estimated as the temporal derivative of the measured YFP concentration corrected for cell growth. The black arrow shows the threshold between active and inactive promoter states defined at two standard deviations from the mean of the non-induced cell distribution (blue). **(C)** Genealogy trees of *mar* promoter activity for different inducer concentrations. The vertical axis represents time in minutes. To binarize the promoter activity we used the threshold from **(A)**. Below the threshold, the *mar* promoter is inactive (blue), above the threshold the promoter is active (yellow, orange, red).

The [YFP] measurements do not give a direct measure of when the promoter is on or off, as the cells grow and thus accumulate and dilute YFP proteins (Figure 3A). We therefore estimated the *mar* promoter activity by monitoring temporal variations of [YFP] while accounting for changes in cell volume due to growth. To simplify our analysis, we used a threshold to binarize *mar* promoter activities as active or inactive within genealogy trees. In the non-induced condition, the *mar* promoter exhibited a natural leakiness (characteristic of most bacterial promoters) that produced a basal level of promoter activity. The threshold was chosen such as to be between one and two standard deviations away from the mean value of the non-induced state (0 mM salicylate) and one standard deviation away from the mean of the highly induced state (3 mM salicylate; Figure 3B). Thus, cells with promoter activities below the threshold had an inactive *mar* promoter, while those with activities above the threshold had an active *mar* promoter. This binarization allowed for simple counting of active cells and inactive cell sub-trees (Figure 3C).

**Figure 4:**
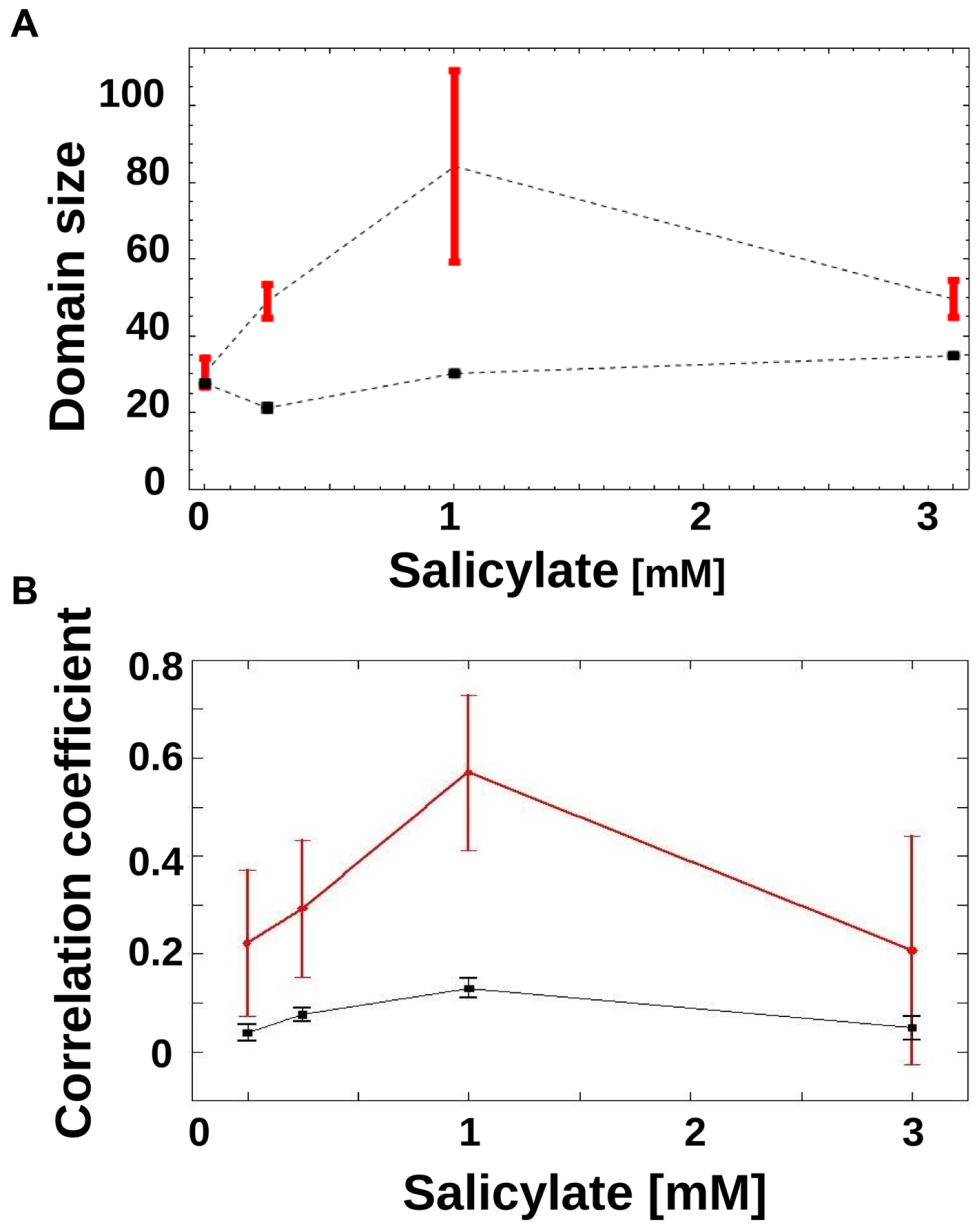
Inheritance of *mar* promoter activity. **(A)** Domain Size of trees is shown for wild-type genealogy trees (circles) and randomly reshuffled genealogy trees (squares) at different inducer concentrations. Error-bars are the standard error. **(B)** Correlations between mother/daughter promoter activity is shown for wild-type genealogy trees (red circles) and randomly reshuffled genealogy trees (black squares) as a function of increasing inducer. Correlation coefficients were calculated for mother/daughter pairs when expression has reached a steady state, in the interval of 120 to 250 min. *N* = 165 (number of mother/daughter pairs) for 0 mM salicylate, *N* = 180 for 0.25 mM salicylate, *N* = 90 for 1 mM salicylate, *N* = 72 for 3 mM salicylate. For reshuffled trees, the activities of each tree were shuffled within the tree itself, to generate 100 instances per tree using the same tree structure. Error-bars are 95% confidence intervals.

As is apparent in Figure 3C, in the 1 mM condition, there is a coexistence of larger sub-trees of active and inactive cells, which form a patchy structure on the genealogy tree. In order to characterize this patchiness that is a signature of heterogeneity, we calculated the product of the average sizes of active and inactive sub-trees in a genealogy tree, and call this domain size (see Materials and methods). As the sub-trees are the result of an underlying inheritance process, the domain size serves as a measure of inheritance. If the average size of either active or inactive sub-trees is close to zero, then the degree of inheritance is small. When both active and inactive sub-trees are large, the degree of inheritance is high. We found that the observed inheritance was highest in genealogy trees with cells induced at intermediate levels (Figure 4A). This measure was insensitive to specific thresholds of promoter activity (Supplementary Figure S2).

A different way of measuring inheritance of promoter activity from mother to daughter cells is to determine the correlation coefficient of the activity, which can also be done without the need to binarize the promoter activity. As can be seen in Figure 4B, the correlation coefficient between mother and daughter promoter activity displays a clear peak at 1 mM, and thus confirms the result obtained with the domain size measure.

### Observed inheritance is not a result of random switching

If promoter activity switches randomly at each cell division, then the activity across trees would exhibit a “speckled” or “salt and pepper” appearance, and thus a noisy distribution. Alternatively, if cells inherit the ancestral promoter activity state, then trees would display large sub-trees of similar promoter activity. A detailed molecular model of *mar* operon induction would leave many parameters free because reaction rates and concentrations, which govern the dynamics of *mar* induction, have been only partially quantitatively characterized (Martin and Rosner, 1995).

To test if random switching alone can explain the observed promoter activity distribution, we generated randomized activity trees and calculated their domain size product. In order to render the theoretical result of the genealogy trees as relevant as possible to the experimentally obtained trees, we constructed each tree by randomly placing active and inactive cells on the leaves of the tree while keeping the number of active and inactive cells identical to those from the empirical genealogy trees. For all non-zero inducer concentrations, the randomized trees had a similar domain size product (Figure 4A, black squares) that was always lower than that of the experimental trees (Figure 4A, red circles). This pattern was robust to specific thresholds of promoter activity (Supplementary Figure S2).

**Figure 5:**
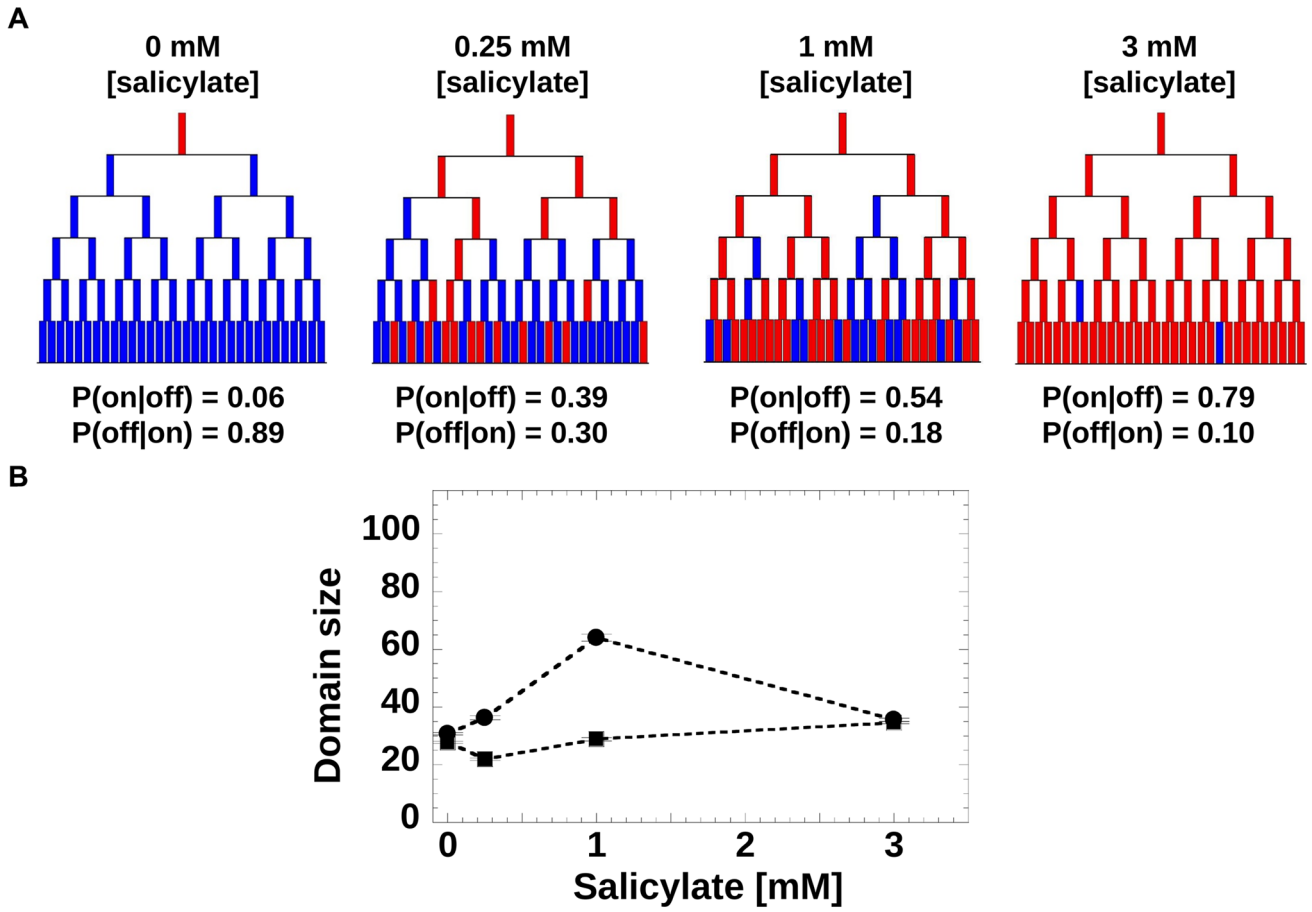
Toy model of *mar* promoter activity. **(A)** Toy model of *mar* operon induction. Cells can switch between active and non-active states with conditional transition probabilities P(on|off) and P(off|on). Values of the probabilities were determined from the experimental data. The genealogy trees follow cells through generations and illustrate switching between an inactive (blue) and active (red) promoter. **(B)** Domain Size product of trees was computed from 200 model simulations using the experimentally-derived conditional probabilities, P(on|off) and P(off|on; circles), and non-conditional probabilities, P(on) and P(off) when the inheritance strength is zero (squares).

Moreover, we asked if the induction patterns observed within genealogy trees could be captured by a simple toy model based on a Markovian stochastic process, wherein the size of the sub-trees is governed by cell switching from “on” to “off” and vice versa. For such a model, we hypothesized that at each generation, a cell has a given probability of switching the *mar* promoter activity of its mother from inactive to active (or from active to inactive). The switching probabilities depend on the inducer concentration and can be inferred directly from experiments (Figure 5A). Using the experimental switching probabilities for each inducer level as parameters for the toy model, we found that in agreement with the experimental trees (Figure 4A), the simulated trees exhibited the highest inheritance for intermediate inducer levels (Figure 5B).

## Discussion

Our experimental technique has shown that phenotypic inheritance is also a measurable quantity in gene regulatory networks with analog output, and not only in bistable genetic networks. We have shown that inheritance may be sensitive to conditions, such as the induction level in the *mar* operon, which remains undetectable when measurements are performed at the population level (Cohen et al., 1993). The standard picture of the transcriptional regulation by MarR and MarA of the *marRAB* and *acrAB* operons has been established mostly through population level experiments (Martin et al., 1996). Based on these experiments we hypothesized that, under steady level of induction, the *mar* promoter from single cells would also exhibit a steady level of activity. Surprisingly, our experimental approach showed that even well after the promoter reaches its steady-state value measured at the population level (~120 min post induction), some single cells had an inactive *mar* promoter. Monitoring promoter activity through multiple generations revealed an unusual pattern of activity in induced cells within each isolated bacterial colony. We found that within lineages, cells with similar promoter activity form sub-trees across generations, suggesting an inducible inheritance where the activity of daughter cells reflects the promoter activity of the mother cell. Surprisingly, the level of inheritance peaks for an intermediate inducer concentration of 1 mM in *E. coli* cells.

This inducible inheritance is more subtle and transient than in all-or-none examples, such as the *ara* and *lac* operons and bacterial persistence and has therefore been more elusive and difficult to trace. Here, by measuring promoter activity across lineages and by simplifying the output by means of binarization, we were able to measure the degree of cell-to-cell inheritance. This technique should be broadly applicable, to a number of gene regulatory networks, using readily available plasmid reporter libraries (Zaslaver et al., 2006), e.g., flagellar assembly cascade (Kim et al., 2020), ribosomal assembly and metabolic networks. Using full lineages instead of solely the mother cell, could be applied at a high-throughput level using promoter libraries, wide-field fluorescence microscopy, and automated image-processing software. Using this type of systems approach could reveal heretofore unknown inheritance phenotypes in the gene regulatory networks of *E. coli* and other single-celled organisms.

While single-cell studies have recently have shown that the mar operon controlled efflux system AcrAB-TolC could be a source of heterogeneous phenotypes (Bergmiller et al., 2017; Meouche and Dunlop, 2018; Wen et al., 2018), the mechanistic origin of the inheritance we uncovered through our experimental and theoretical analysis remains an open question. For one, the intricate network of negative and positive feedbacks that the *marRAB* and *acrAB* operons form (Figure 1E) is by its very topological construction prone to complex dynamics behavior. What adds extra complexity to this already rich dynamics, is the recently discovered biased partitioning of the AcrAB-TolC pumps at old poles (Bergmiller et al., 2017), which in itself is also an inheritance effect. Thus, maybe what we observe here could be a superposition of two types of inheritance mechanisms and quantitatively unraveling the contribution of each will be an experimentally and theoretically challenging task. Lastly, that salicylate induces inheritance effects in the activity of the *mar* operon should pique the interest of clinicians who assess the effects of aspirin on the *mar* operon induction in microbiome bacterial residents.

## Data availability statement

The raw data supporting the conclusions of this article will be made available by the authors, without undue reservation.

## Author contributions

CCG and PC designed the research. CCG, LB, and PO performed the research. CCG, LB, PO, MA, and PC analyzed the data. CCG, LB, PC and PO wrote the paper. All authors contributed to the article and approved the submitted version.

## Funding

This work was supported by NIH P50 award P50GM081892-02 to the University of Chicago, a catalyst grant from the Chicago Biomedical Consortium with support from The Searle Funds at The Chicago Community Trust to PC, and a Yen Fellowship to CCG. MA was partially supported by PAPIIT-UNAM grant IN-11322.

## Conflict of interest

The authors declare that the research was conducted in the absence of any commercial or financial relationships that could be construed as a potential conflict of interest.

## Publisher’s note

All claims expressed in this article are solely those of the authors and do not necessarily represent those of their affiliated organizations, or those of the publisher, the editors and the reviewers. Any product that may be evaluated in this article, or claim that may be made by its manufacturer, is not guaranteed or endorsed by the publisher.
