# Supplementary material for: Monitoring lineages of growing and dividing bacteria reveals an inducible memory of mar operon expression
Source: Front Microbiol. 2023 Jun 20;14:1049255. doi: 10.3389/fmicb.2023.1049255 (PMC10359894; doi:10.3389/fmicb.2023.1049255)
Supplement: Supplementary file 1 [file Presentation_1.PPTX]

## Slide 1
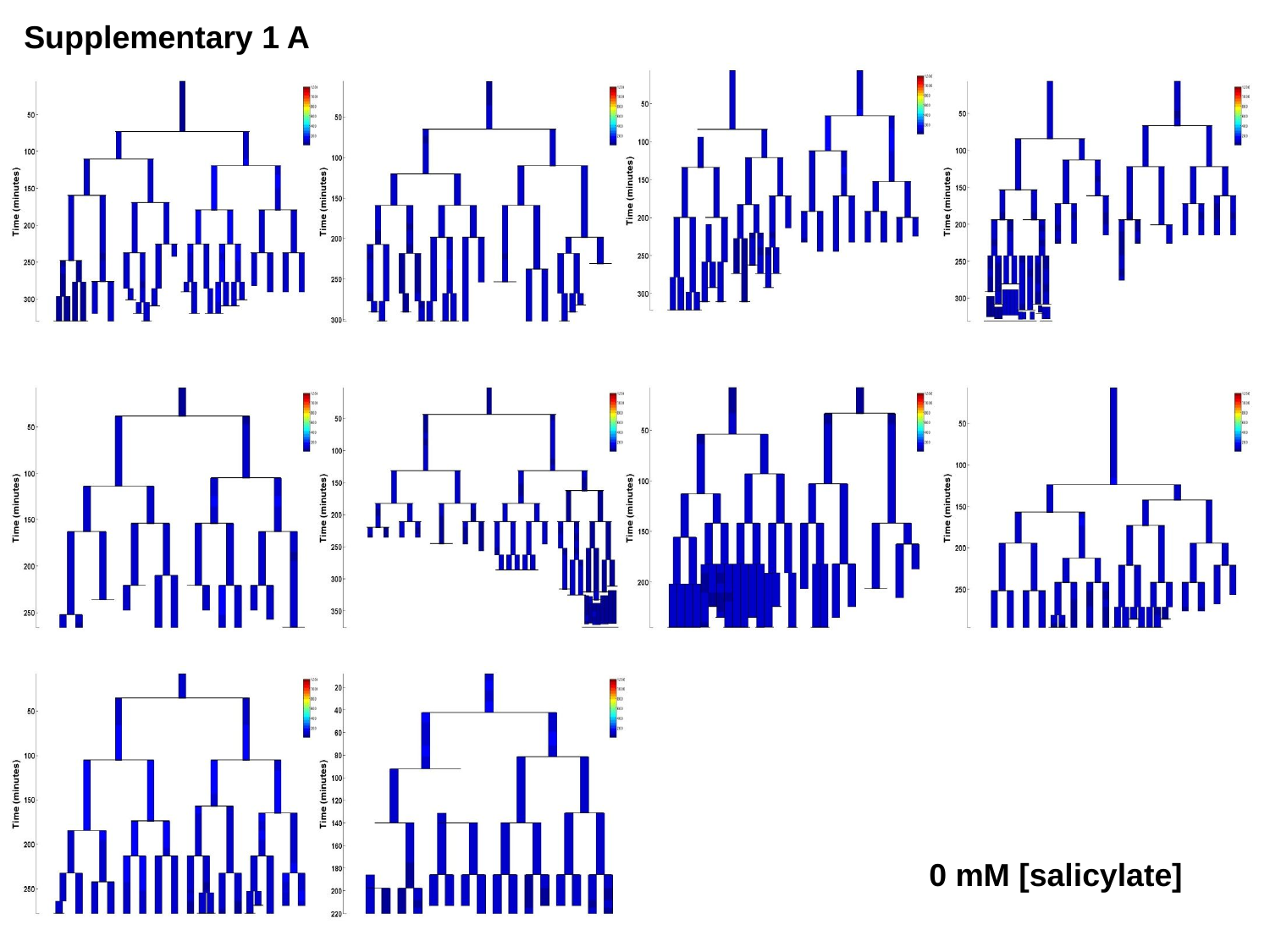

Supplementary 1 A
0 mM [salicylate]

## Slide 2
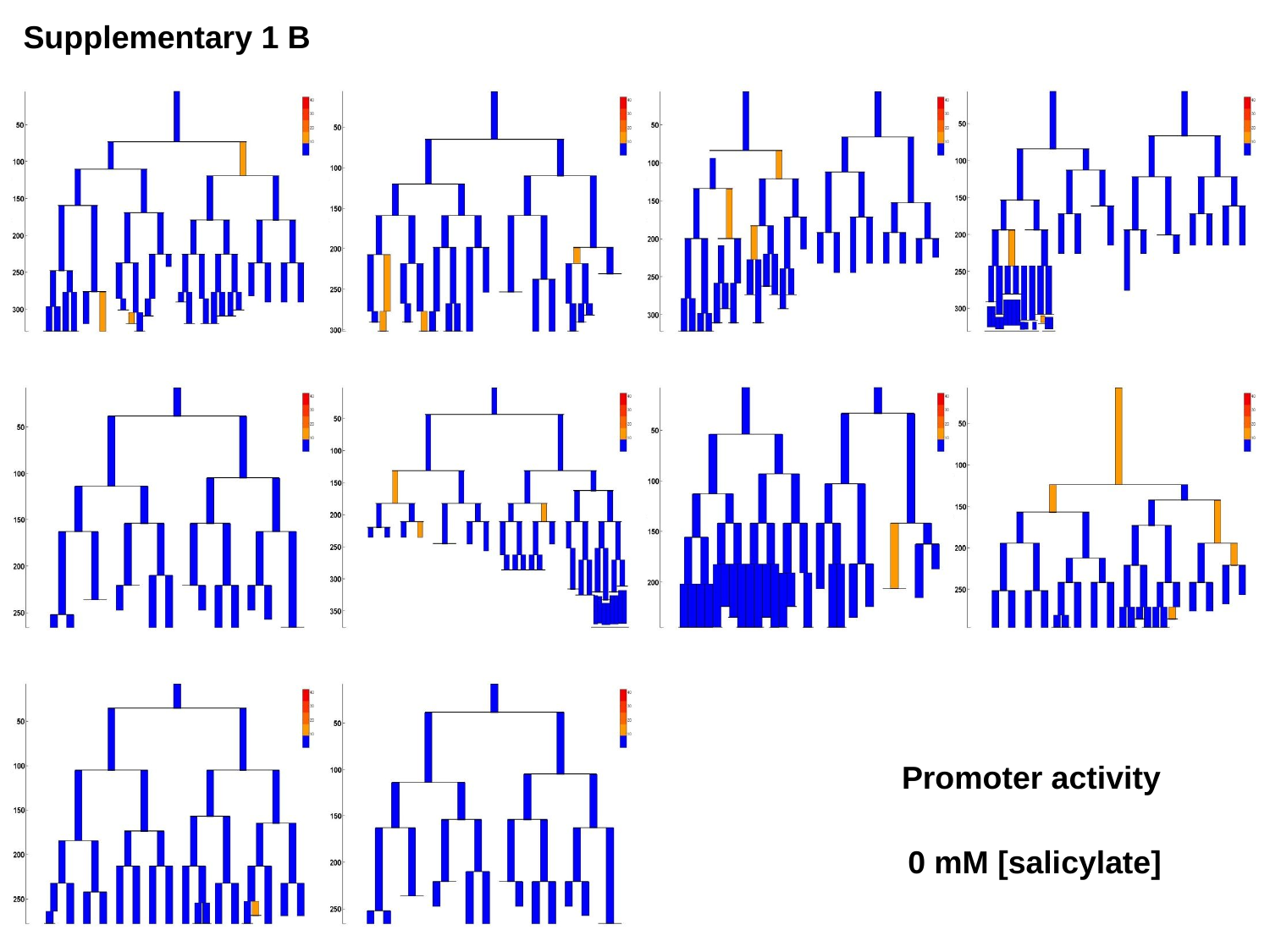

Supplementary 1 B
Promoter activity
0 mM [salicylate]

## Slide 3
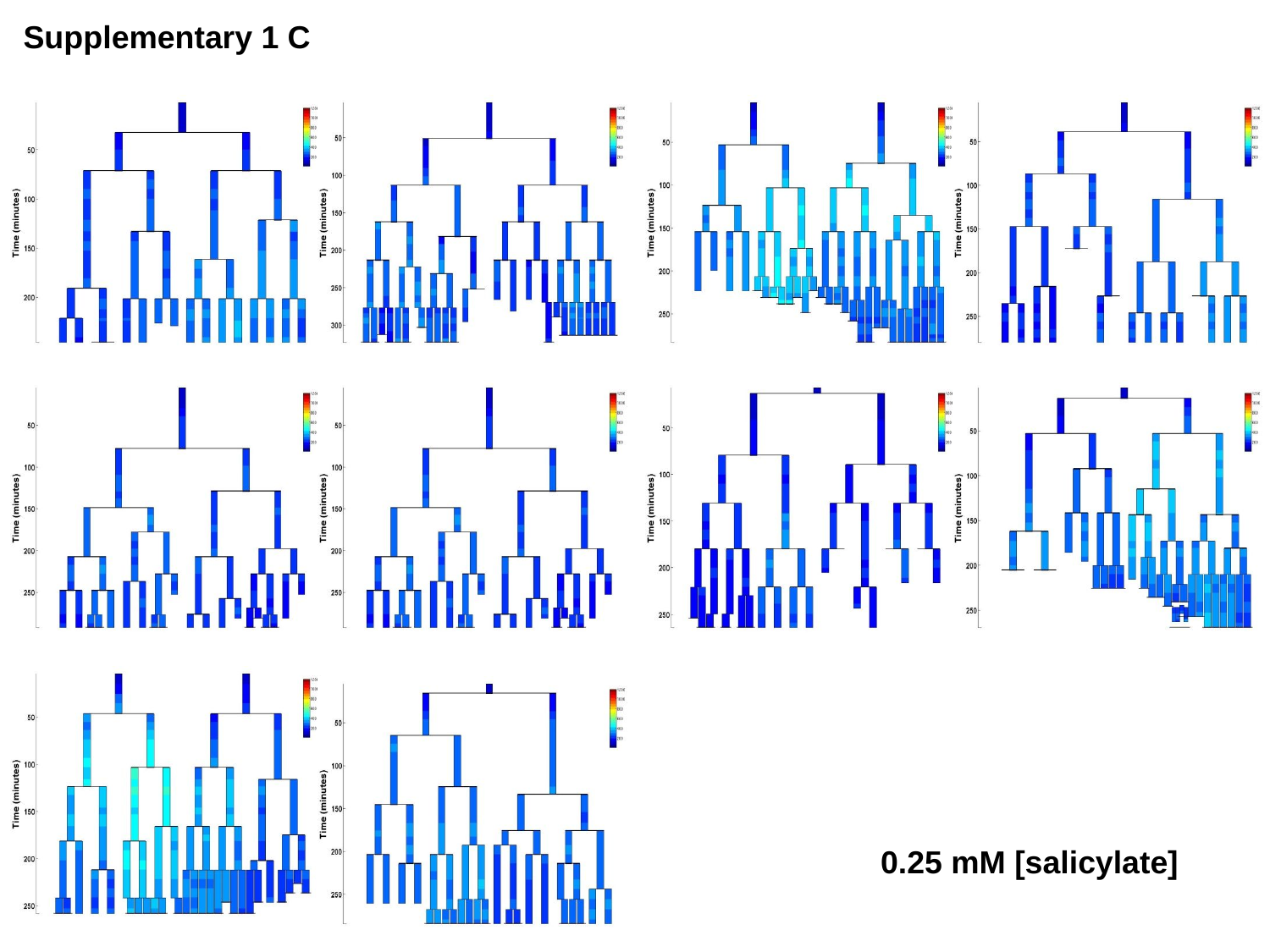

Supplementary 1 C
0.25 mM [salicylate]

## Slide 4
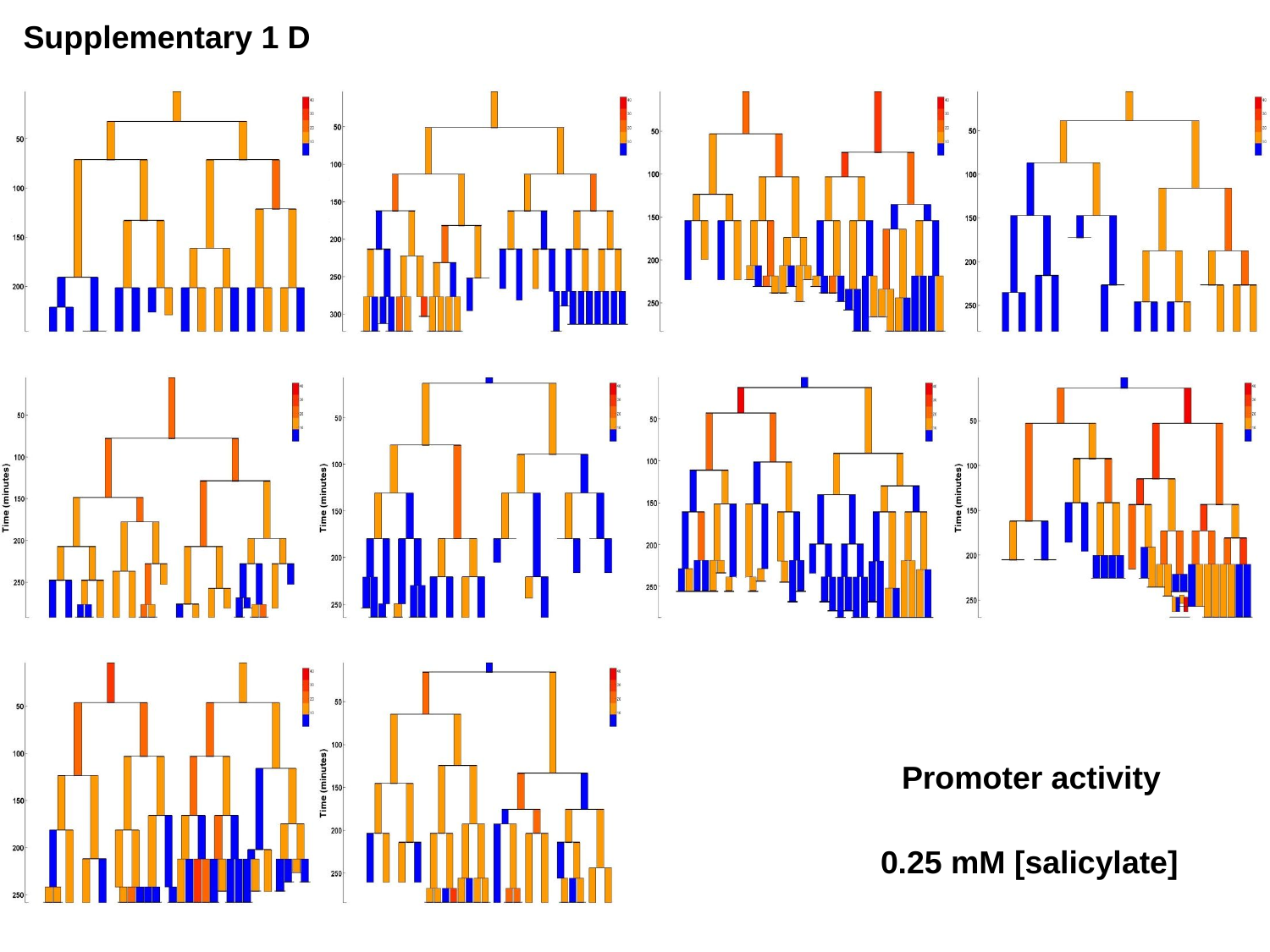

Supplementary 1 D
Promoter activity
0.25 mM [salicylate]

## Slide 5
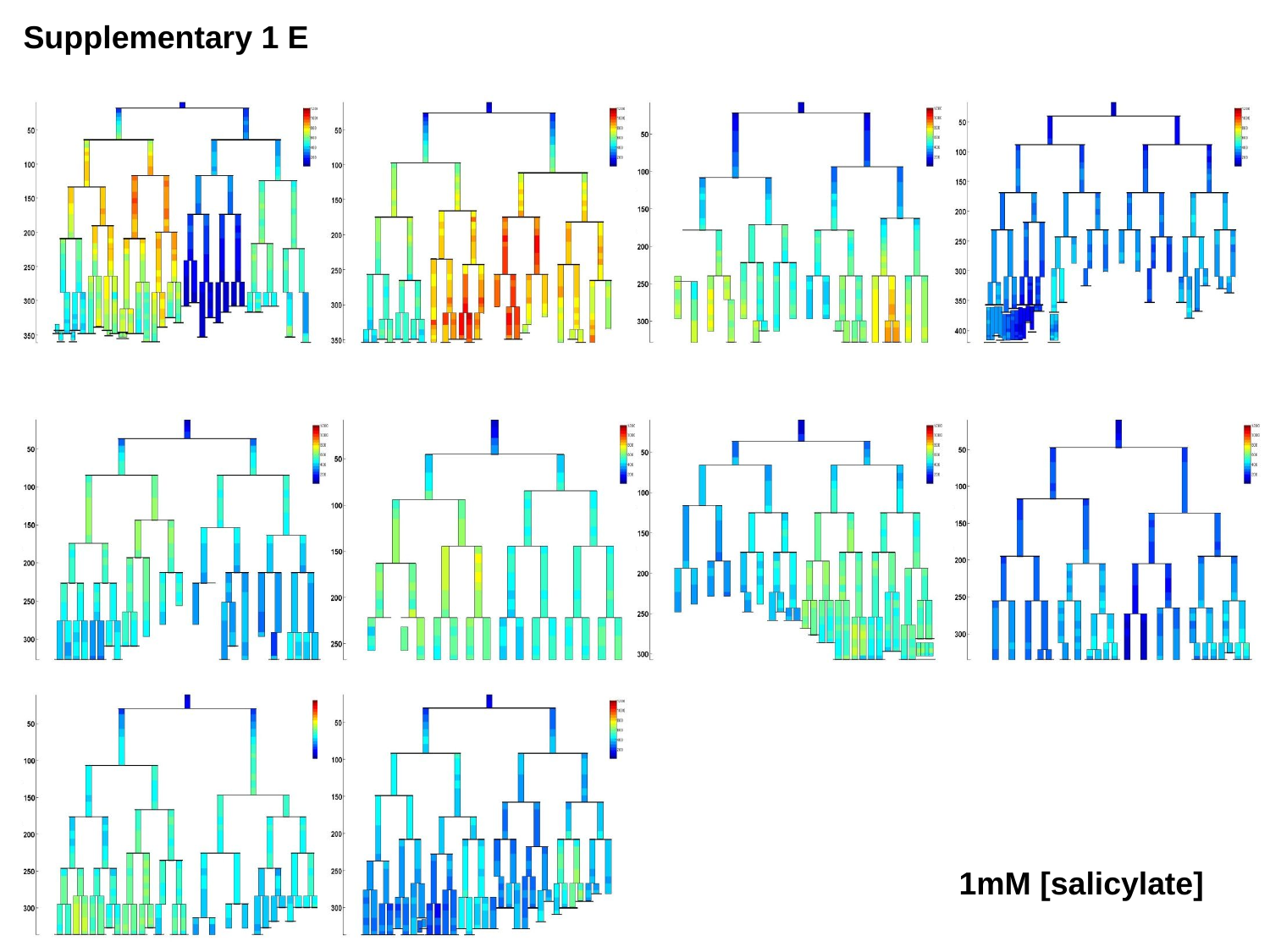

Supplementary 1 E
1mM [salicylate]

## Slide 6
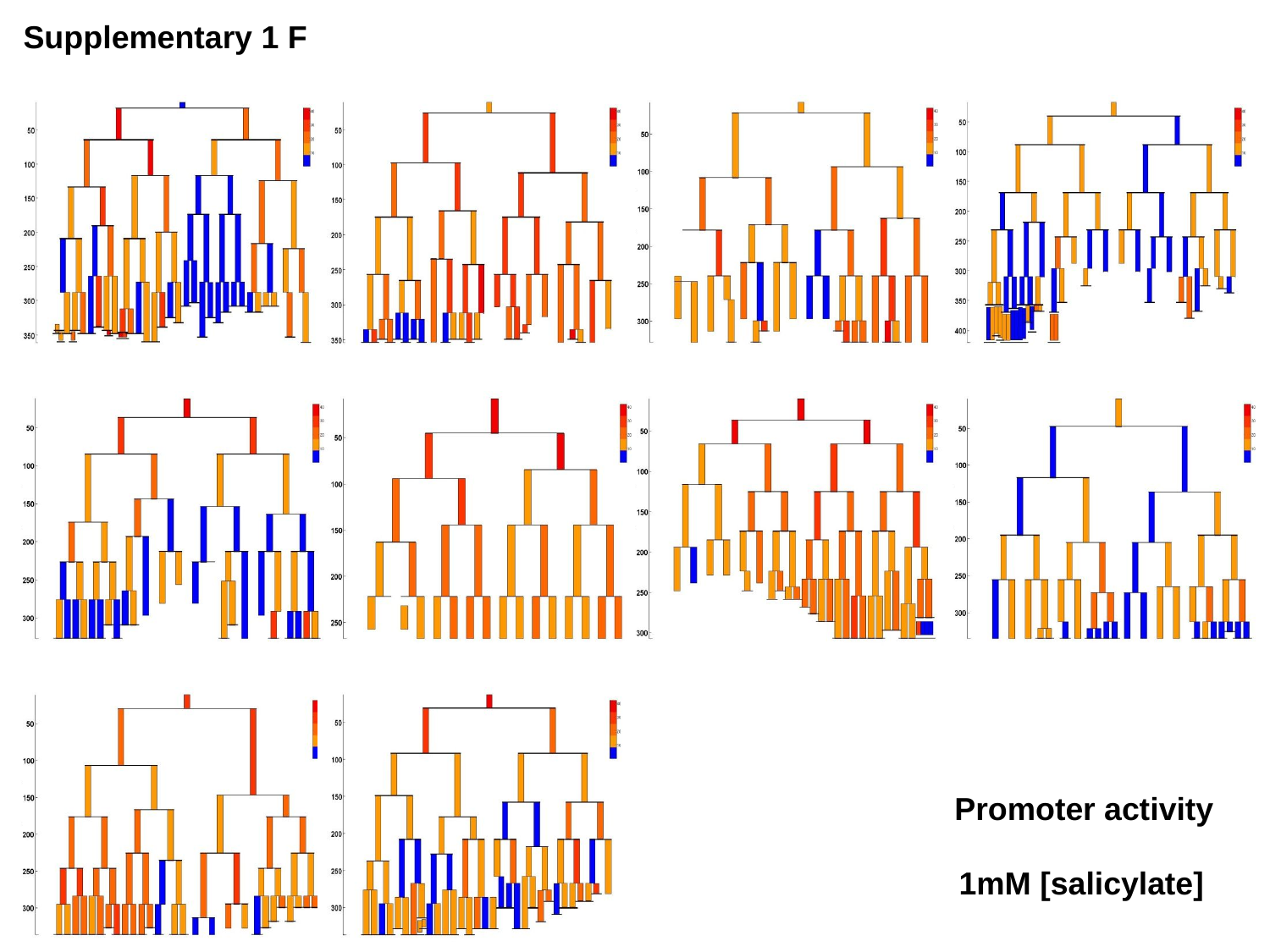

Supplementary 1 F
Promoter activity
1mM [salicylate]

## Slide 7
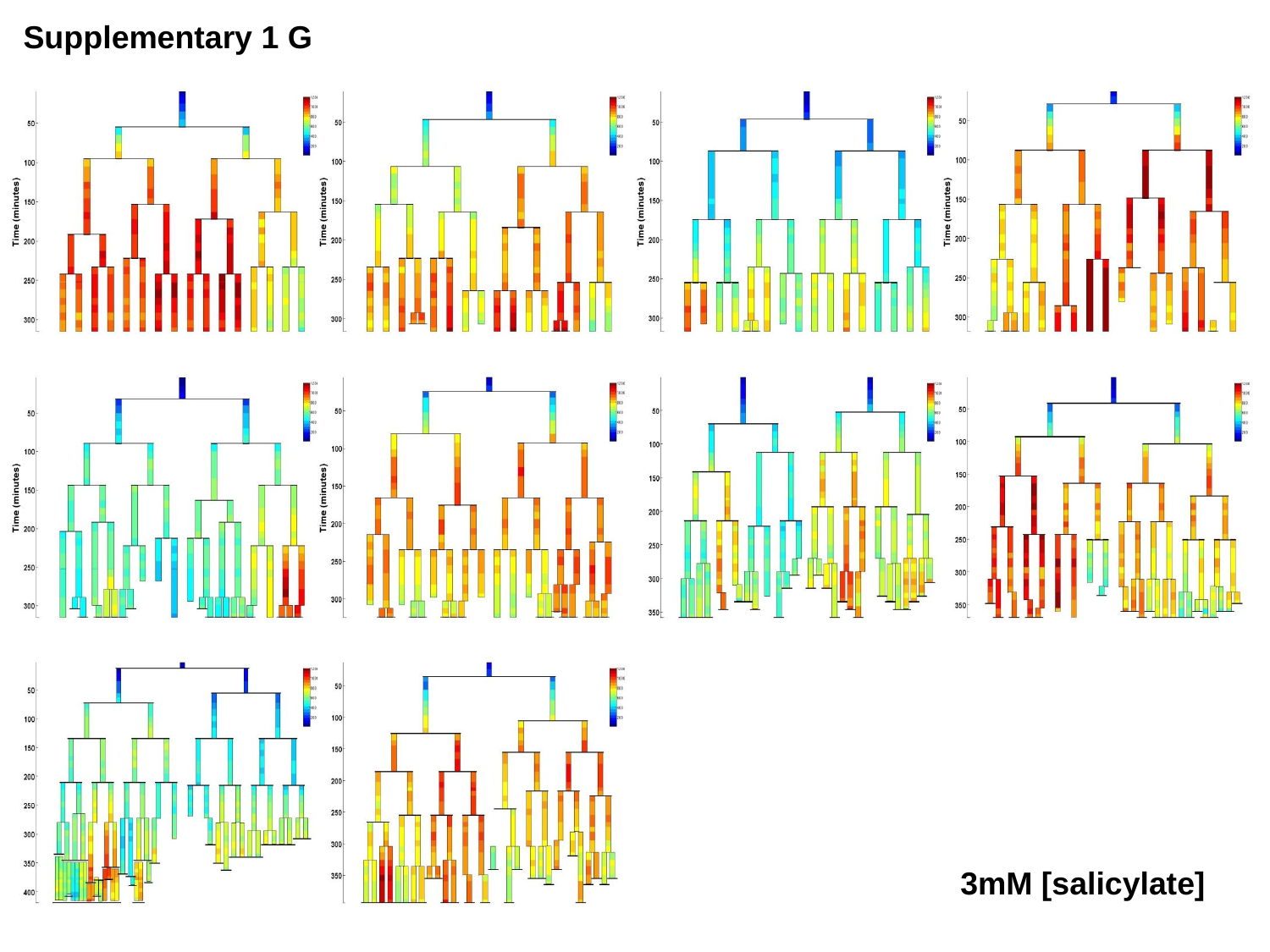

Supplementary 1 G
3mM [salicylate]

## Slide 8
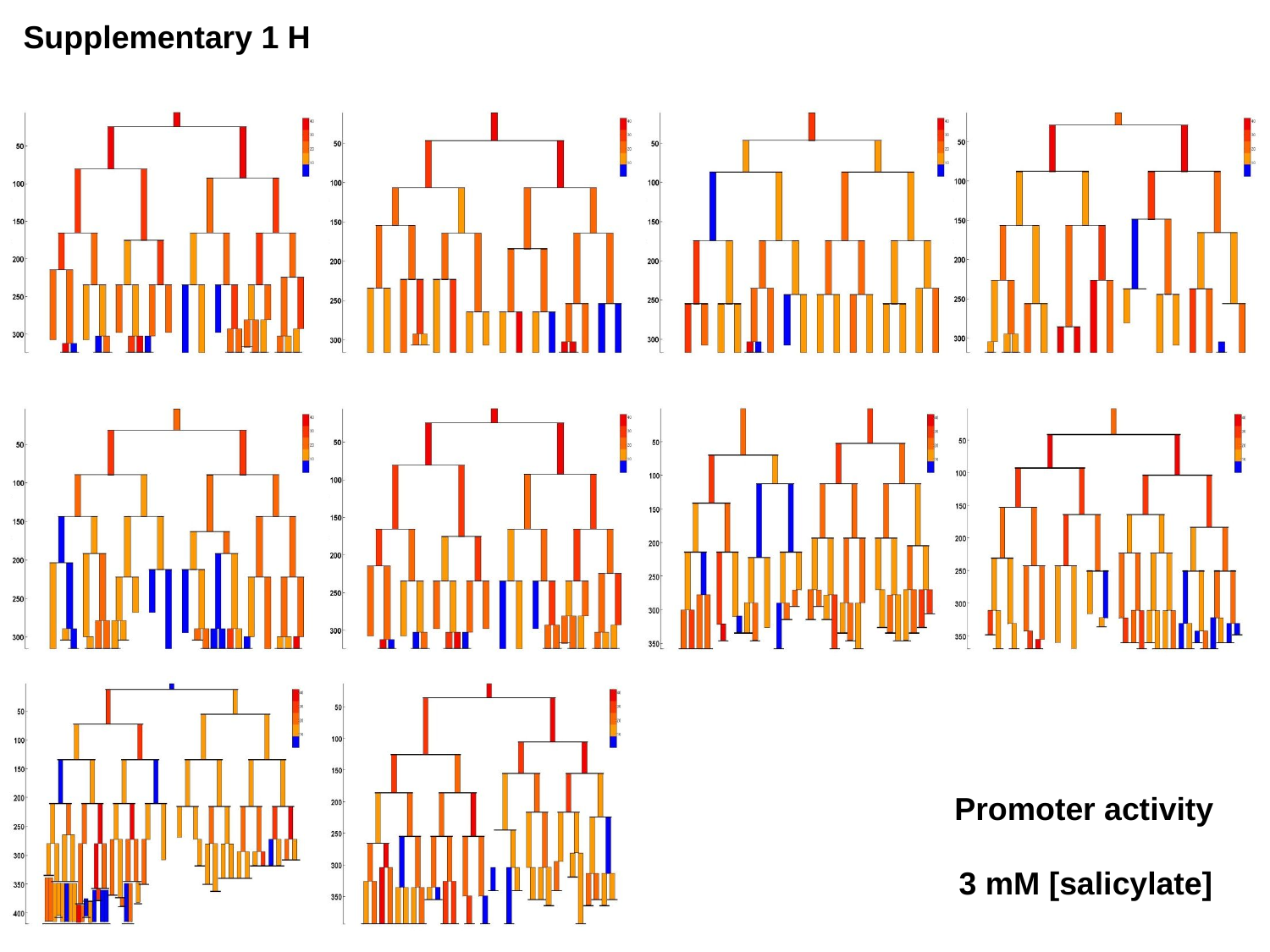

Supplementary 1 H
Promoter activity
3 mM [salicylate]

## Slide 9
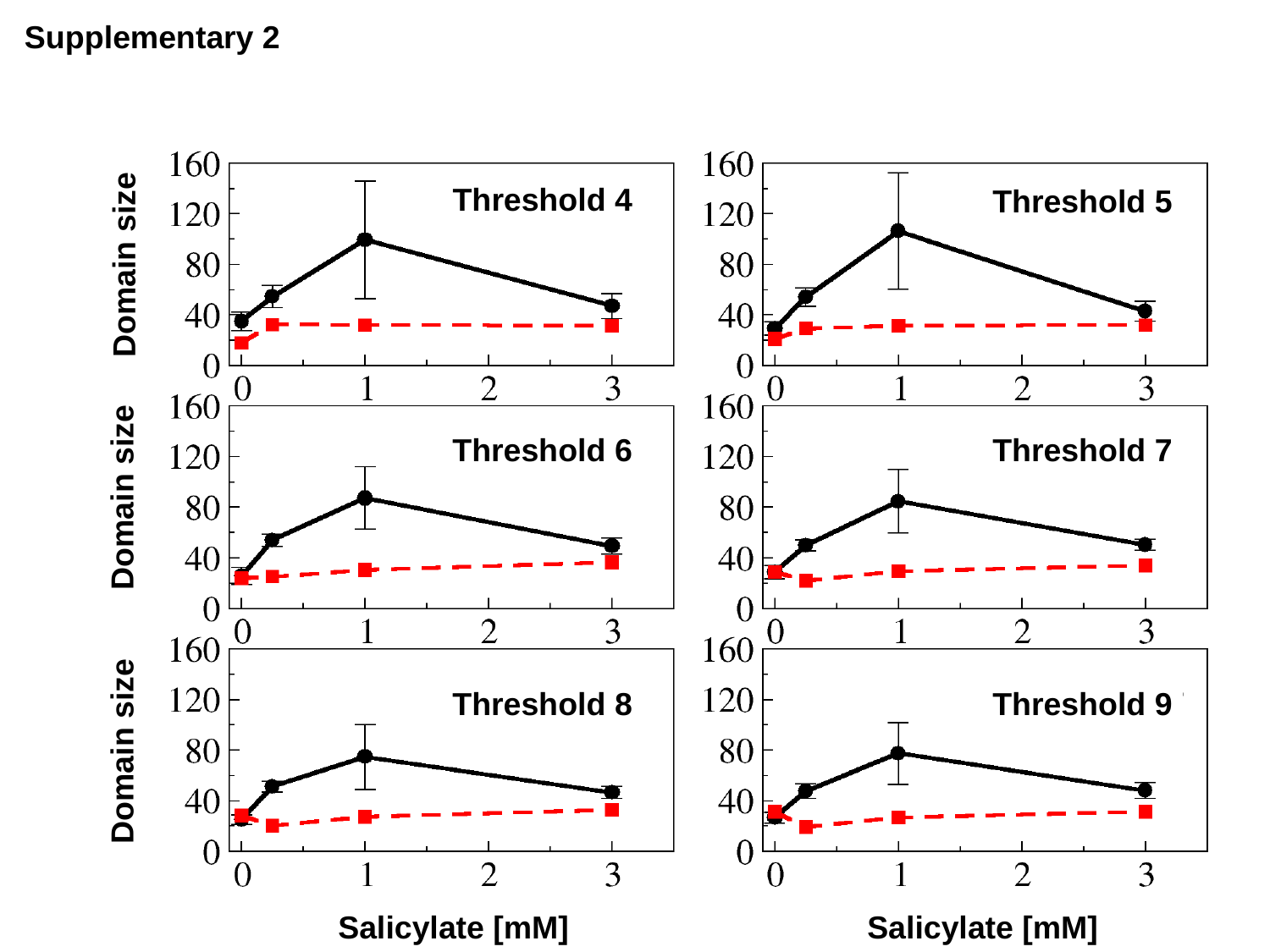

Supplementary 2
Threshold 4
Threshold 5
Domain size
Threshold 6
Threshold 7
Domain size
Threshold 9
Threshold 8
Domain size
Salicylate [mM]
Salicylate [mM]
